# AMP-activated protein kinase contributes to zinc-induced neuronal death via activation by LKB1 and induction of Bim in mouse cortical cultures

**DOI:** 10.1186/s13041-016-0194-6

**Published:** 2016-02-09

**Authors:** Jae-Won Eom, Jong-Min Lee, Jae-Young Koh, Yang-Hee Kim

**Affiliations:** Department of Molecular Biology, Sejong University, Seoul, 143-747 South Korea; Department of Integrative Bioscience and Biotechnology, Sejong University, Seoul, 143-747 South Korea; Neural Injury Research Lab & Department of Neurology, University of Ulsan College of Medicine, Seoul, 138-736 South Korea

**Keywords:** AMPK, Brain ischemia, LKB1, Bim, Caspase-3, Neuronal death, Zinc

## Abstract

**Background:**

We reported that zinc neurotoxicity, a key mechanism of ischemic neuronal death, was mediated by poly ADP-ribose polymerase (PARP) over-activation following NAD^+^/ATP depletion in cortical cultures. Because AMP-activated protein kinase (AMPK) can be activated by ATP depletion, and AMPK plays a key role in excitotoxicity and ischemic neuronal death, we examined whether AMPK could be involved in zinc neurotoxicity in mouse cortical neuronal cultures.

**Results:**

Compound C, an AMPK inhibitor, significantly attenuated zinc-induced neuronal death. Activation of AMPK was detected beginning 2 h after a 10-min exposure of mouse cortical neurons to 300 μM zinc, although a significant change in AMP level was not detected until 4 h after zinc treatment. Thus, AMPK activation might not have been induced by an increase in intracellular AMP in zinc neurotoxicity. Furthermore, we observed that liver kinase B1 (LKB1) but not Ca^2+^/calmodulin-dependent protein kinase kinase β (CaMKKβ), was involved in AMPK activation. Although STO-609, a chemical inhibitor of CaMKKβ, significantly attenuated zinc neurotoxicity, zinc-induced AMPK activation was not affected, which suggested that CaMKKβ was not involved in AMPK activation. Knockdown of LKB1 by siRNA significantly reduced zinc neurotoxicity, as well as zinc-induced AMPK activation, which indicated a possible role for LKB1 as an upstream kinase for AMPK activation. In addition, mRNA and protein levels of Bim, a pro-apoptotic Bcl-2 family member, were noticeably increased by zinc in an AMPK-dependent manner. Finally, caspase-3 activation in zinc-induced neuronal death was mediated by LKB1 and AMPK activation.

**Conclusions:**

The results suggested that AMPK mediated zinc-induced neuronal death via up-regulation of Bim and activation of caspase-3. Rapid activation of AMPK was detected after exposure of cortical neuronal cultures to zinc, which was induced by LKB1 activation but not increased intracellular AMP levels or CaMKKβ activation. Hence, blockade of AMPK in the brain may protect against zinc neurotoxicity, which is likely to occur after acute brain injury.

## Background

Zinc serves as a component of zinc finger proteins and DNA-binding proteins that are important for the regulation of genes [1] and enzyme activity in all cells [2]. Like other tissues, the brain has high levels of intracellular zinc, but free zinc levels in the cytoplasm are very low because most zinc ions are tightly bound to macromolecules, such as proteins and nucleic acids [3–5]. Furthermore, the brain has an additional pool of free zinc in a subset of synaptic vesicles [6]. Although the function of synaptic zinc is still under investigation, recent evidence suggests that synaptic zinc is released into the synaptic cleft where it induces synaptic activity [7–9]. However, during acute brain injury caused by stroke, epilepsy, or brain trauma, an excess amount of zinc is released, which enters into post-synaptic neurons via calcium permeable routes, and leads to neuronal cell death [3, 10]. In addition, oxidative stress may increase intracellular free zinc by inducing release from intracellular stores such as lysosomes, and from zinc-bound proteins such as metallothioneins, leading to neuronal death [11, 12]. Many key players, such as PKC, NADPH oxidase, nNOS, PARP, and caspase are involved in zinc-induced neuronal death [13–16], and processes such as oxidative stress, necrosis [17, 18], apoptosis [18–20], and lysosomal membrane permeabilization (LMP) [21] are related to zinc neurotoxicity.

AMP-activated protein kinase (AMPK), a serine/threonine kinase, is critical for the homeostasis of cellular energy in most eukaryotic tissues. Diverse physiological stressors induce ATP consumption that leads to an increase in the intracellular AMP/ATP ratio, which results in increased AMPK activity [22, 23]. Activated AMPK inhibits energy-consuming biosynthetic pathways, such as gluconeogenesis, fatty acid synthesis, protein synthesis, and cell proliferation, which are necessary to maintain energy homeostasis [22–24].

Functional AMPK is a hetero-trimeric complex that consists of a catalytic subunit (α) and two regulatory subunits (β and γ). Several isomers of each subunit have been reported (α1, α2; β1, β2; γ1, γ2, γ3) [22, 23]. The α-subunit has a kinase domain that can be phosphorylated by AMPK kinase itself, which increases its enzymatic activity, whereas regulatory subunits simply facilitate the phosphorylation or dephosphorylation of the catalytic subunit via conformational change [22, 23].

There are two well-known upstream AMPK kinases: liver kinase B1 (LKB1; also known as serine/threonine kinase 11 [STK11]) and Ca^2+^/calmodulin-dependent protein kinase kinase β (CaMKK β) [24–26]. Although the mechanisms of activation of these two kinases are different, LKB1 controls phosphorylation through the AMP-mediated pathway, whereas CaMKK β controls the Ca^2+^-mediated pathway [23–25]; LKB1 and CaMKK β work together to activate AMPK [25]. In addition, LKB1 is expressed in nearly every tissue [24, 27] and its deletion results in considerable, albeit incomplete, inhibition of AMPK activity [28]. CaMKK β functions in specific tissues such as neurons [29], T-cells [30], and endothelial cells [31]. The functional LKB1 complex, which consists of LKB1 and two accessory subunits (STRAD and MO25), phosphorylate threonine 172 (T172) of AMPK. Thus, modulation of the LKB1-STRAD-MO25 complex regulates AMPK activity via differential accessibility to phosphatase activity [22, 23, 25], which indicates that LKB1 complex acts as a master upstream kinase for AMPK [25].

Because AMPK is an energy sensor, and energy depletion can lead to ischemic stroke, several studies have examined the involvement of AMPK in brain ischemia. In fact, numerous studies showed that over-activation of AMPK mediated ischemic brain injury [26, 32–34]. In addition, brain ischemia was shown to result in neuronal cell death through necrosis and apoptosis [18], which are processes that require ATP [35]; inhibition of AMPK activity attenuated neuronal cell death [33]. Some *in vivo* studies demonstrated that deletion of AMPK α2, but not α1, was protective against ischemic brain injury [32, 33]; a similar result was reported for the heart [28], which suggested a deleterious role for AMPK α2 in ischemic stroke. Moreover, Concannon et al. [36] showed that excitotoxicity via *N*-methyl-D-aspartic acid (NMDA) receptors led to neuronal cell death by disruption of ion homeostasis, which was mediated by over-activation of AMPK and induction of the pro-apoptotic Bcl-2 homology domain 3 (BH3)-only protein, Bim [27].

Our previous study has shown that during zinc-induced neuronal death, over-activation of poly(ADP-ribose) polymerase-1 (PARP-1) almost completely consumed the intracellular pool of NAD^+^/ATP [16], which triggered AMPK activation in zinc neurotoxicity. Therefore, in the present study, we examined whether AMPK played a critical role in zinc neurotoxicity, and which upstream and downstream signaling molecules were involved in AMPK-mediated zinc neurotoxicity.

## Results

### AMPK activation was involved in zinc neurotoxicity

**Fig. 1:**
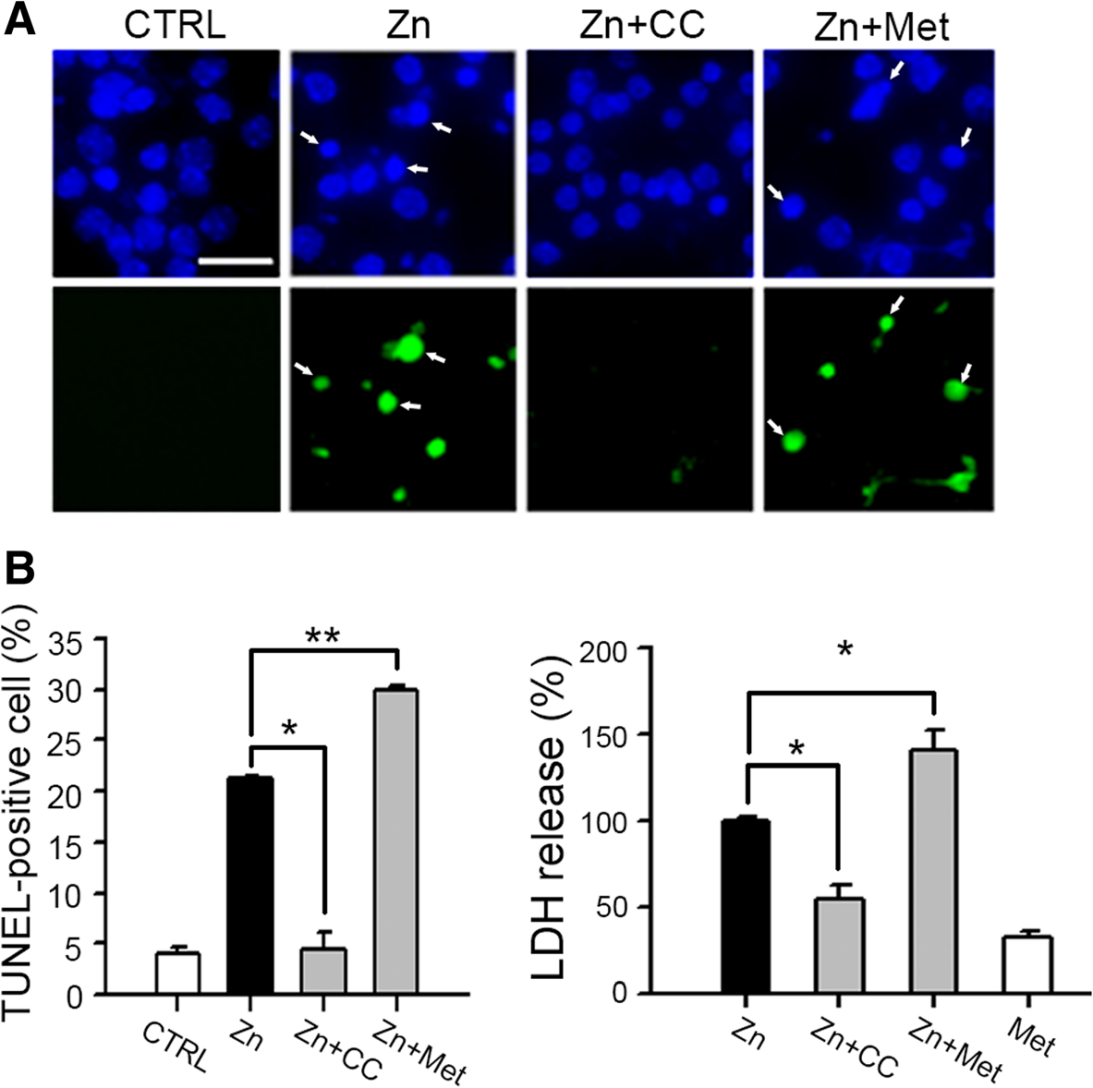
Requirement for AMPK in zinc neurotoxicity. **a** Microscopic images of Hoechst 33342-stained total nuclei (upper) or TUNEL-positive apoptotic nuclei (lower) of the same field in mouse cortical near-pure neuronal cultures at 12 h after 10 min exposure to 300 μM zinc with or without 20 μM Compound C (CC) or 50 μM metformin (Met). Arrows indicate apoptotic shrunk and fragmented nuclei. Scale bar, 25 μm. **b** Quantitative data for TUNEL-positive apoptotic cells (left, mean ± SEM, *n* = 3 cultures) or LDH release (right, mean ± SEM, *n* = 4 cultures) in near-pure neuronal cultures after 12 h exposure of 300 μM zinc for 10 min with or without CC or Met, or metformin alone. *p < 0.05 and **p < 0.01 compared to zinc-exposed cultures, two-tailed *t*-test

Studies have shown that AMPK was involved in cytotoxicity or cell death [26, 36–39], although contrary opinions exist [26, 40]. Several research groups showed that excitotoxicity or ischemic brain injury in a rat model was significantly attenuated by inhibition of AMPK activity [34, 36, 41, 42], which suggested a role for AMPK in neurotoxicity. Many research groups have determined that zinc-induced neuronal death is one neurotoxic mechanism that underlies ischemic brain injury [10, 43–48]; therefore, we investigated the involvement of AMPK in zinc-induced neuronal death. Nearly-pure mouse neuronal cultures were exposed to zinc (300 μM for 10 min) along with the selective AMPK inhibitor, compound C (20 μM) or the AMPK activator, metformin (50 μM). Here, we observed significantly fewer TUNEL-positive cells (*P* < 0.05) (Fig. 1a, indicated by arrow) in samples treated with the AMPK inhibitor, compound C, compared to samples treated with zinc alone. Conversely, samples treated with the AMPK activator, metformin, had a significantly increased number of apoptotic cells (*P* < 0.01) (Fig. 1a). LDH release, a consequence of cell death, was significantly decreased by compound C (*P* < 0.05) but increased by metformin (*P* < 0.05) (Fig. 1b). These findings suggested the involvement of AMPK activation in zinc-mediated neurotoxicity.

### Zinc-induced AMPK activation was not mediated by increased intracellular AMP level

**Fig. 2:**
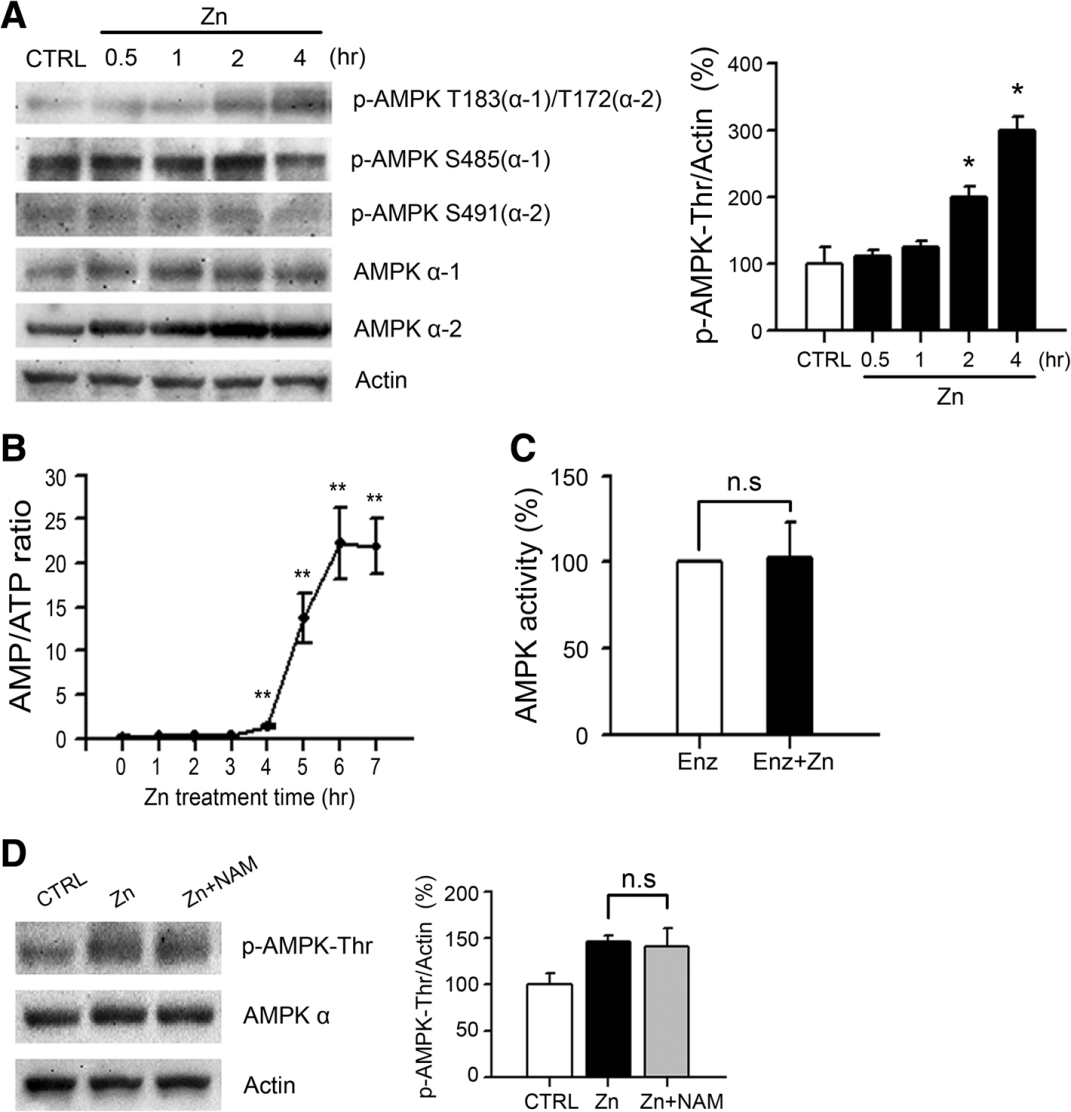
AMP-independent activation of AMPK in zinc neurotoxicity. **a** Representative western blot (left) and quantitation (right) results to document phosphorylation of AMPK over the time-course of zinc treatment. Protein samples were prepared from near-pure neuronal cultures at the indicated time points after 10 min exposure to 300 μM zinc. Actin was used as loading control. Graph data depict the mean ± SEM levels of AMPK phosphorylation of threonine residues (T183 in α-1/T172 in α-2) normalized to actin and are expressed as a percent of control levels (*n* = 3/group, *p < 0.05 compared to sham controls, two-tailed *t*-test). Whereas noticeable AMPK phosphorylation on serine residues was not detected, AMPK phosphorylation on threonine residues (T183 in α-1/T172 in α-2) was detected beginning 2 h after zinc treatment. **b** Intracellular AMP/ATP ratio over the time-course of zinc treatment (300 μM for 10 min). A significant increase in AMP/ATP ratio was detected beginning 4 h after zinc treatment. **p < 0.01 compared to sham controls, two-tailed *t*-test. **c** AMPK enzyme activity assay performed using recombinant AMPK-α2 protein with or without zinc (1 μM) (mean ± SEM, *n* = 4). *In vitro* AMPK enzyme activity was not directly affected by zinc treatment. **d** Representative western blots and quantitation of the phosphorylation of AMPK (*n* = 3/group). Protein samples were prepared from near-pure neuronal cultures at 2 h after 300 μM zinc for 10 min with or without 2 mM nicotinamide (NAM), a chemical inhibitor of PARP. The activation of AMPK at 2 h was not attenuated by NAM

Because we observed that AMPK activation was required for zinc neurotoxicity, we examined whether the phosphorylation of AMPK was induced by zinc in near-pure cortical cultures. The major phosphorylation site of AMPK, threonine 183/172 (T183 in AMPK α-1 or T172 in AMPK α-2), and an additional candidate site, serine 485/491 (S485 in AMPK α-1 or S491 in AMPK α-2) [49], were targeted by western blotting. We detected clear phosphorylation on T183/172 beginning 2 h after zinc treatment (300 μM for 10 min), whereas we observed no measurable phosphorylation on S485 or S491 (Fig. 2a). In addition, we found a zinc-triggered increase of expression levels for both AMPK α-1 and α-2 (Fig. 2a) from a very early time point, 30 min, after zinc treatment.

Previously, we showed that zinc-induced neuronal death was mediated by NAD^+^/ATP depletion in context of PARP over-activation [16]. Thus, we analyzed changes in the intracellular AMP/ATP ratio as a trigger for AMPK activation using a time-course experiment in near-pure neuronal cultures exposed to zinc. Interestingly, we found that zinc-induced AMPK activation was not triggered by the increase of intracellular AMP levels, because the AMP/ATP ratio increased significantly beginning at 4 h post zinc treatment, which reflected either significant ATP depletion or significant AMP increase (*P* < 0.01) (Fig. 2b) compared to AMPK activation beginning 2 h after zinc treatment (Fig. 2a). It is generally acknowledged that AMPK senses the intracellular AMP/ATP ratio and then is activated by an increase of AMP/ATP ratio; therefore, these results indicated that AMPK activation by zinc was independent from the intracellular AMP levels, and this activation might require another signaling modulator.

To begin, we tested whether zinc directly affected the activation of AMPK. Zinc chloride was added directly to purified, recombinant AMPK α-2, and then *in vitro* AMPK activity was measured (Fig. 2c). The result of this experiment showed that AMPK was not directly activated by zinc.

Next, we confirmed that PARP-1 over-activation was involved in AMPK activation by zinc. The early activation of AMPK at 2 h after zinc treatment was not attenuated by nicotinamide (NAM), a chemical inhibitor of PARP-1, which suggested that the initial phosphorylation of AMPK in zinc-induced neuronal death was not mediated by PARP-1. Taken together, these results indicated that rapid activation of AMPK in zinc neurotoxicity was not induced by PARP-1 over-activation or intracellular AMP/ATP ratio increase.

### Identification of LKB1 as an upstream kinase for AMPK activation in zinc neurotoxicity

**Fig. 3:**
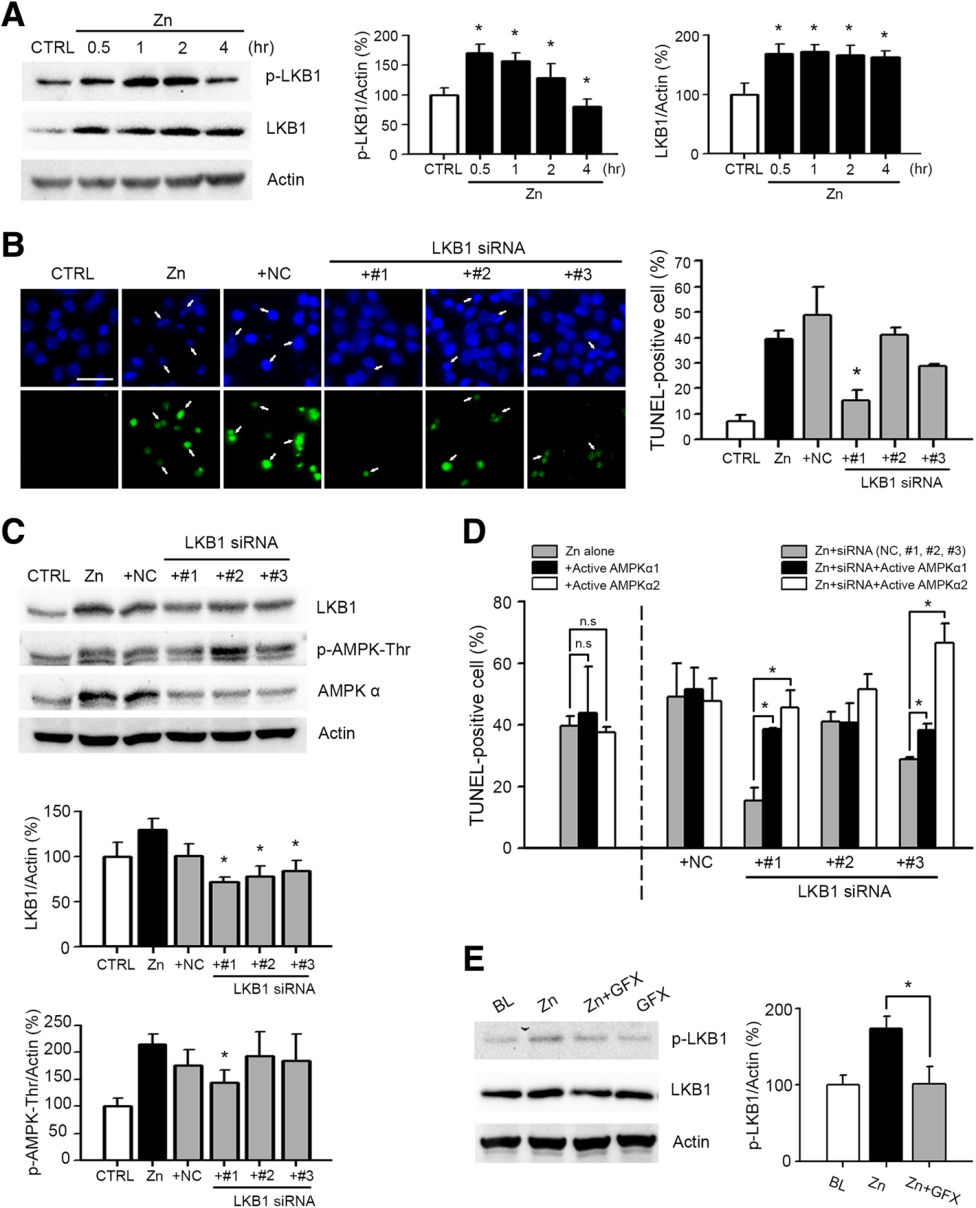
Possible role of LKB1 as an upstream kinase of AMPK in zinc neurotoxicity. **a** Representative western blots and quantitation of the phosphorylation and expression of LKB1 over the time-course of zinc treatment (*n* = 3/group, *p < 0.05 compared to sham controls, two-tailed *t*-test). Protein samples were prepared from zinc-treated (300 μM for 10 min), near-pure neuronal cultures at the indicated time points. **b** Microscopic images (left) and quantitative data (right, mean ± SEM, *n* = 3 cultures) of Hoechst 33342-stained total nuclei (upper) and TUNEL-positive apoptotic nuclei (lower) of the same field in mouse cortical near-pure neuronal cultures at 9 h after a 10-min exposure to 300 μM zinc with or without siRNA knockdown with three different LKB1 siRNAs (#1, #2 or #3); scrambled siRNA was the negative control (NC). Arrows indicate apoptotic nuclei. Scale bar, 25 μm. *p < 0.05 compared to zinc-exposed cultures, two-tailed *t*-test. **c** Representative western blots and quantitation of the expression levels of LKB1 and phosphorylation of AMPK (*n* = 3/group, *p < 0.05 compared to zinc-exposed cultures). Near-pure cortical neuronal cultures were transfected with siRNAs at DIV3 for 48 h, and then excess zinc was added (300 μM for 10 min). Protein samples were prepared at 3 h after zinc treatment. **d** Quantitative data for TUNEL-positive apoptotic cells (mean ± SEM, *n* = 4 cultures) in mouse cortical near-pure neuronal cultures at 9 h after a 10-min exposure to 300 μM zinc with or without siRNA alone or siRNA plus cDNA of active form of human AMPKα1 or AMPK α2. Introduction of the active form of AMPKα1/α2 reversed neuroprotection by LKB1 siRNA #1 and significantly increased neuronal death in LKB1 siRNA #3 knockdown condition. **e** Representative western blots and quantitation of phosphorylation and expression levels of LKB1 (*n* = 3/group, *p < 0.05 compared to zinc-exposed cultures). Protein samples were prepared from cortical neuronal cultures at 0.5 h after 300 μM zinc for 10 min with or without 5 μM GF109264X (GFX), a chemical inhibitor of PKC. The phophorylation and expression levels of LKB1 induced by zinc were reversed by PKC inhibition

Because we observed that an increase in the intracellular AMP/ATP ratio was not the trigger for AMPK activation in zinc neurotoxicity, we needed to identify, which upstream signaling molecules were involved in AMPK activation in zinc-induced neuronal death. LKB1 is a tumor suppressor, often mutated in human Peutz-Jeghers syndrome, and a well-known upstream kinase of AMPK [25, 50]. Because LKB1 directly phosphorylates AMPK on T172 in an AMP-dependent manner [22, 25], we examined the possibility that LKB1 mediated AMPK activation after zinc treatment. Initially, we observed that expression of LKB1 and phosphorylated LKB1 were increased beginning 30 min after zinc treatment (Fig. 3a), which correlated with a very early time point in comparison with AMPK activation at 2 h (Fig. 2a). In addition, we observed that the level of phosphorylated LKB1 returned to baseline at 4 h after zinc treatment, while the expression level of LKB1 was sustained up to 4 h after zinc treatment (Fig. 3a).

Next, we examined whether LKB1 knockdown reduced zinc-induced AMPK activation and neurotoxicity. In primary cortical neuronal cultures, the efficiency of transfection with siRNA was very low; despite this problem, we continued to use siRNA for LKB1 knockdown because there are no specific chemical inhibitors for LKB1. Thus, three different sequences of LKB1 siRNA (#1, #2, and #3) were transfected into near-pure neuronal cultures, and then treated with zinc (300 μM for 10 min). Although all three siRNAs significantly attenuated the expression levels of LKB1 in mouse cortical neuronal cultures (Fig. 3c), only one siRNA (LKB1 siRNA#1) significantly reduced the number of TUNEL-positive apoptotic nuclei (indicated by arrows) (*P* < 0.05) (Fig. 3b). Consistent with this result, the levels of phosphorylated AMPK were significantly decreased only by siRNA #1 (Fig. 3c). To confirm the effect of LKB1 knockdown on zinc-mediated AMPK phosphorylation and neurotoxicity, we next examined whether the introduction of the active form of AMPK into LKB1 knockdown cells could rescue the neuroprotective effects of LKB1 knockdown on zinc-induced neuronal death. Confirming the possibility of an LKB1-AMPK axis, neuroprotection by siRNA #1 was completely reversed by co-transfection with the active form of human AMPKα1 or AMPKα2, and slight reduction of neuronal death by siRNA#3 was also significantly diminished by co-expression of the active form of AMPK (Fig. 3d). Collectively, these results suggested that LKB1 was able to activate AMPK as an upstream signaling molecule in zinc neurotoxicity.

Many studies showing the LKB1-AMPK signaling pathway have been reported, but the upstream regulatory mechanism for LKB1 is not fully understood. LKB1 has several autophosphorylation sites in the C-terminal region, *i.e.,* Ser31, Thr185, Thr189, Ser325, Thr336, Thr363, Thr402, and Ser428 [51]. Among these, it has been shown that Thr363 is phosphorylated by ATM in response to ionizing radiation [52], and Ser428 is phosphorylated by cAMP-dependent kinase (PKA), p90RSK, and protein kinase C zeta (PKCζ) [53–55]. Because we previously reported that zinc-induced neuronal death was mediated by PKC activation in mouse cortical cultures [14, 16], we next tested the possibility that phosphorylation of LKB1 was mediated by PKC in zinc neurotoxicity. Supporting the involvement of PKC in LKB1 phosphorylation, zinc-induced LKB1 phosphorylation was almost completely attenuated by GF109203X, a chemical inhibitor of PKC (Fig. 3e). Taken together, rapid PKC activation by zinc may be an upstream signal for LKB1 and AMPK activation in zinc-mediated neuronal death.

### CaMKKβ was not an upstream kinase for zinc-induced AMPK activation

**Fig. 4:**
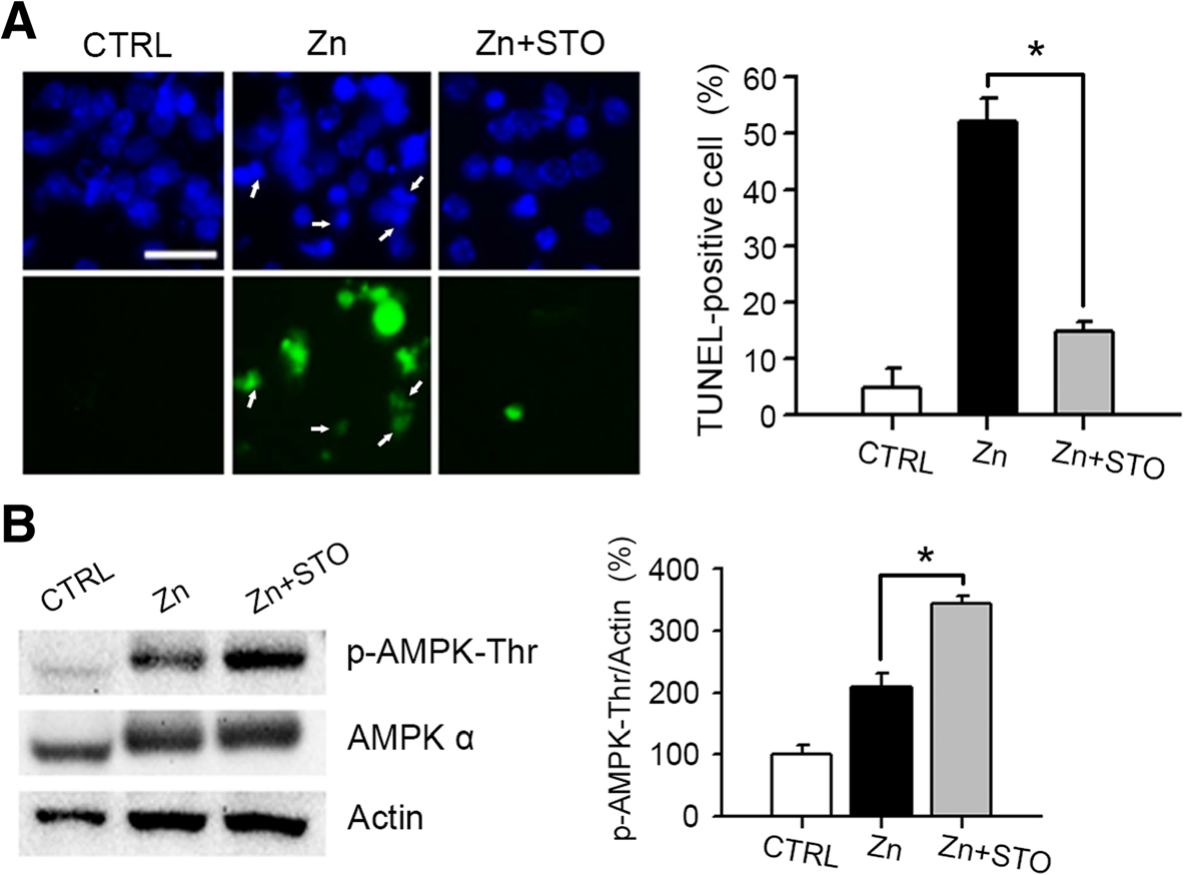
CaMKKβ as an upstream kinase of AMPK was not involved in zinc neurotoxicity. **a** Microscopic images (left) and quantitative data (right, mean ± SEM, *n* = 3 cultures) of Hoechst 33342-stained total nuclei (upper) and TUNEL-positive apoptotic nuclei (lower) of the same field in mouse cortical near-pure neuronal cultures at 12 h after a 10-min exposure to 300 μM zinc with or without 10 μM STO-609 (STO), a CaMKKβ inhibitor. Arrows indicate apoptotic nuclei. Scale bar, 25 μm. *p < 0.05 compared to zinc-exposed cultures, two-tailed *t*-test. Zinc-induced neuronal cell death was markedly attenuated by STO. **b** Representative western blots and quantitation of phosphorylated AMPK (*n* = 3/group, *p < 0.05). Protein samples were prepared from near-pure neuronal cultures at 4 h after 300 μM zinc treatment for 10 min with or without 10 μM STO-609. Phosphorylation of AMPK was not reduced by STO-609

CaMKKβ, which activates AMPK via a Ca^2+^-dependent pathway, is another well-known upstream kinase of AMPK [25, 56]. Treatment of near-pure neuronal cultures with STO-609 (10 μM), a selective CaMKKβ antagonist, significantly reduced zinc-induced neuronal death (*P* < 0.05) (Fig. 4a). However, we confirmed by western blotting that AMPK activation was not attenuated by the CaMKKβ inhibitor (Fig. 4b). In fact, AMPK phosphorylation was significantly increased by the inhibition of CaMKKβ (Fig. 4b). Therefore, although CaMKKβ may be involved in zinc neurotoxicity, AMPK activation was not mediated by CaMKKβ in zinc neurotoxicity.

### AMPK activation mediated the induction of Bim and the resultant activation of caspase-3 in zinc neurotoxicity

**Fig. 5:**
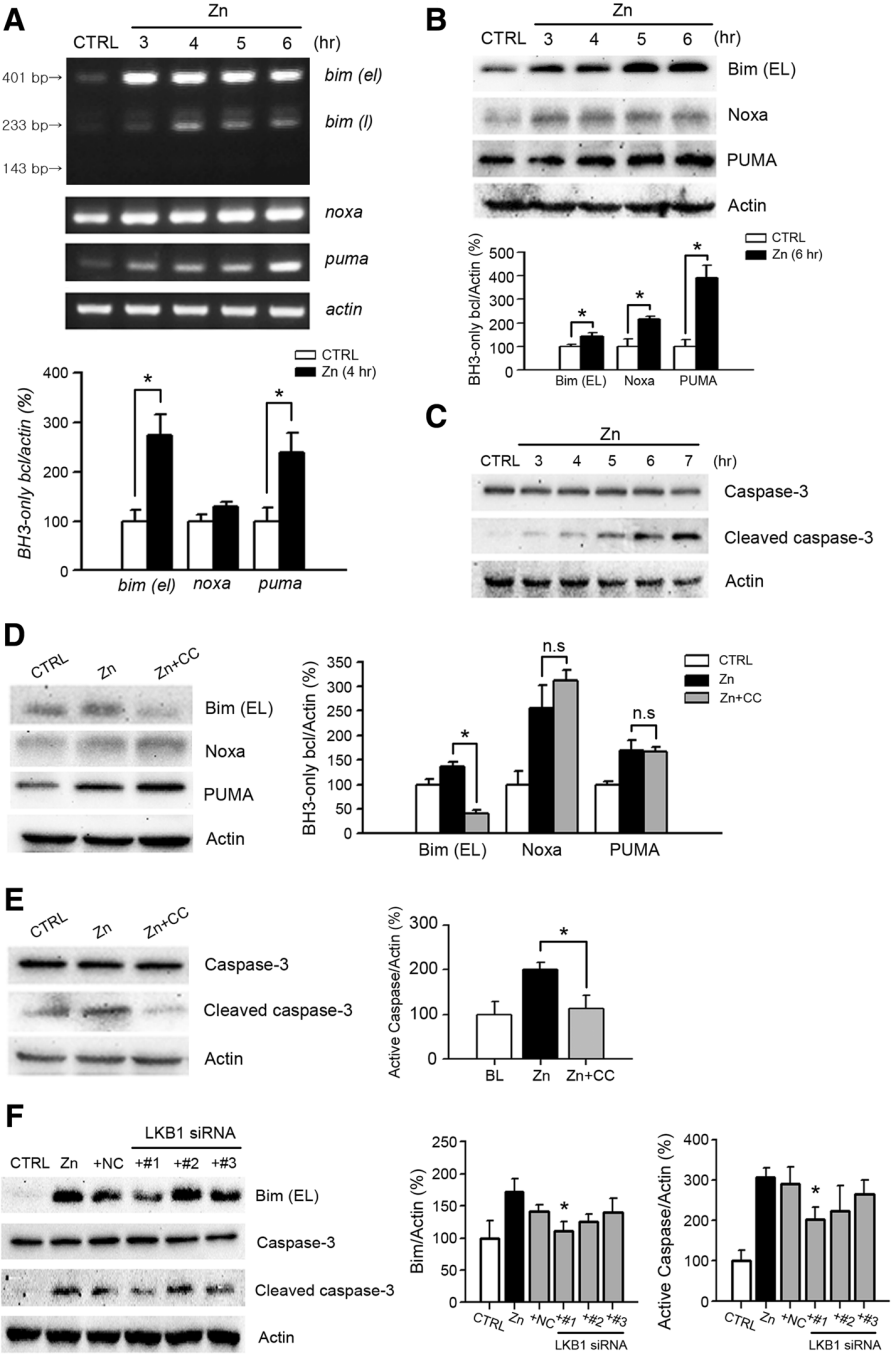
AMPK-mediated induction of Bim, and resultant activation of caspase-3 in zinc neurotoxicity. (**a** and **b**) RT-PCR (**a**) and western blots (**b**) to measure the expression levels of Bim, Noxa, and Puma over the time-course of zinc treatment. mRNA and protein samples were prepared at the indicated time points from sham-washed or zinc-treated near-pure neuronal cultures (300 μM for 10 min). Graphed data depict the mean ± SEM levels of Bim/Noxa/PUMA normalized to actin at 4 h or 6 h after zinc treatment (*n* = 3/group, *p < 0.05 compared to sham control). Noticeable increases in protein levels of Bim, Noxa, and PUMA were observed beginning 3–4 h after zinc treatment. **c** Western blots for caspase-3 activation. Protein samples were prepared at the indicated time points from near-pure neuronal cultures after zinc treatment. Caspase-3 activation by zinc was clearly observed beginning 5 h after zinc treatment. (**d** and **e**) Representative western blots and quantitation of the expression of Bim, Noxa, and Puma (**d**) and activation of caspase-3 (**e**) (*n* = 3/group, *p < 0.05 compared to zinc-exposed cultures). Protein samples were prepared from near-pure neuronal cultures at 4 h for BH3-only Bcl-2 family proteins (**d**) or 5 h for caspase-3 activation (E) after 10-min exposure to 300 μM zinc with or without 20 μM compound C (CC). Among the BH3-only Bcl-2 family, only the expression of Bim (induced by zinc treatment) was markedly attenuated by Compound C. Caspase-3 activation was also reduced by Compound C in zinc-induced neuronal cell death. **f** Representative western blots and quantitation of the expression of Bim and activation of caspase-3 (*n* = 3/group, *p < 0.05). Near-pure cortical neuronal cultures were transfected with siRNAs for LKB1 at DIV3 for 48 h, followed by the addition of excess zinc (300 μM for 10 min). Protein samples were prepared at 4 h after zinc treatment. LKB1 siRNAs #1 noticeably attenuated Bim induction and caspase-3 activation by zinc treatment

Previously, another group showed that excitotoxicity was mediated specifically by the induction of Bim expression and resultant caspase-3 activation in an AMPK-dependent manner [36]. Therefore, we examined whether the BH3-only Bcl family was involved in AMPK-mediated zinc-induced neuronal death. First, using reverse-transcription (RT)-PCR, we observed that mRNA levels of *bim* and *puma* were noticeably increased 3–4 h after zinc treatment (Fig. 5a). In the case of *noxa*, mRNA levels were also slightly augmented by zinc, but a significant increase was not detected (Fig. 5a). However, western blot analysis showed that the protein levels of Noxa as well as Bim and PUMA were noticeably increased by zinc (Fig. 5b), suggesting the possible activity of the BH3-only Bcl-2 family in zinc-induced neuronal apoptosis. Consistently, clear activation of caspase-3 was observed 5 h after zinc exposure (Fig. 5c). However, the increase in expression among all members of the BH3-only Bcl-2 family may not have been mediated by AMPK, because only Bim was remarkably attenuated by compound C, a potent AMPK inhibitor, and Noxa, and PUMA were not (Fig. 5d). Furthermore, caspase-3 activation by zinc was significantly blocked by Compound C, suggesting the critical role of AMPK in caspase-3 activation by zinc (Fig. 5e). We also observed a reduction of Bim expression and inhibition of caspase-3 by LKB1 siRNA #1 transfection of near-pure neuronal cultures (Fig. 5f). Consistent with the data showing cytotoxicity and AMPK phosphorylation, only LKB1 siRNA #1, but not siRNA #2 and #3, significantly attenuated the induction of Bim and activation of caspase-3 (Fig. 5f). Collectively, these results strongly suggested that the induction of Bim that was mediated by LKB1 and AMPK activation contributed to zinc-induced caspase-3 activation and neurotoxicity.

## Discussion

The main finding of this study was that the LKB1, AMPK, and Bim signaling cascade contributed to zinc-induced caspase-3 activation and neuronal death in mouse cortical neuronal cultures. Although we observed that CaMKKβ played a role in zinc neurotoxicity, CaMKKβ was not an upstream kinase for AMPK activation in zinc-induced neuronal death. Furthermore, neither over-activation of PARP-1 nor increased intracellular AMP levels triggered AMPK activation in zinc-induced neuronal death in mouse cortical neuronal cultures.

Acute brain injury, including ischemic neuronal damage, is related to diverse stressors, such as energy depletion, hypoxia, oxidative stress, acidosis, and calcium and zinc overload [57, 58]. In particular, loss of glucose and oxygen supply alters the AMP/ATP balance, which leads to the critical AMPK activation in the brain [34]. However, the overall consequence of AMPK activation seems to be dependent upon the degree of AMPK activation, cell types activated, and the metabolic status of cells and tissues [41]. Some reports demonstrate that mild activation of AMPK using a low dose of 5-Aminoimidazole-4-carboxamide ribonucleotide (AICAR) attenuates hypoglycemic stress and excitotoxicity in hippocampal neuronal cultures [40] and ceramide-mediated-apoptosis in astrocyte cultures [59]. In addition, the glucose and oxygen concentrations in the culture conditions may alter the status of AMPK activation and regulation [60, 61]. Under physiological glucose (3 mM) and oxygen (5 %) culture conditions, AMPK activation was reset at a new set point, which could reproduce physiological conditions more closely [62, 63]. More recently, other reports have shown the detrimental role of AMPK activation *in vitro* in cell lines and primary cortical neurons, and *in vivo* ischemic injury animal models [34]. In support of these findings, AMPK α2 deletion but not AMPK α1 deletion in transgenic mice significantly reduced infarct volumes after ischemic injury compared to wild-type mice [32]. In another series of experiments, whereas compound C, an AMPK inhibitor, attenuated infarct size and behavioral defects of ischemic injury [33], metformin, an anti-diabetic drug and AMPK activator, exacerbated ischemic injury [64]. More interestingly, chronic metformin improved brain ischemic injury by reducing AMPK activation [64], suggesting that the duration and degree of AMPK activation are critical for cellular responses to ischemia. Although the exact mechanism remains unclear, AMPK activation is detrimental in stroke, and AMPK inhibition is neuroprotective.

Initially, AMPK was known to be activated by an elevated AMP/ATP ratio and related stimuli including exercise, starvation, hypoxia, cellular pH, and redox status [24]. However, many physiological and pathological conditions that do not alter the AMP/ATP ratio modulate AMPK signaling by direct or indirect effects on its kinase activity [65]. Several upstream kinases, such as LKB1 and CaMKKβ, can activate AMPK via phosphorylation at Thr183(α-1)/Thr172(α-2) of the catalytic α subunit [24–26]. Here, we observed that initial activation of AMPK at an early time point after zinc exposure of neurons was not mediated by a change in intracellular AMP/ATP ratio (Fig. 2). Instead, LKB1 activation and induction were critical for AMPK activation. We observed that phosphorylation of LKB1 was preceded by phosphorylation of AMPK (Fig. 3a & Fig. 2a), and showed that knockdown of LKB1 using siRNA noticeably attenuated AMPK activation (Fig. 3c) and zinc neurotoxicity (Fig. 3b). Furthermore, the reduction of zinc neurotoxicity by LKB1 siRNA #1 and #3 was almost completely reversed by co-transfection of the active form of AMPKα1 or AMPKα2 (Fig. 3d), confirming LKB1 as the upstream kinase for AMPK. We also tested whether CaMKKβ may be an upstream kinase for AMPK activation in zinc-induced neuronal death. Although chemical inhibition of CaMKKβ by STO-609 significantly attenuated zinc neurotoxicity, CaMKKβ did not appear to be an upstream kinase for AMPK activation, because the phosphorylation of AMPK was not affected by STO-609. Previously, we demonstrated that zinc-induced neuronal death shows apoptotic and necrotic aspects in cortical cultures [18], and many compounds are involved in zinc toxicity, including PKC, NADPH oxidase, nNOS, PARP-1, ERK, and Egr-1 [13, 14, 16, 66]. Several reports showed that PKCζ may be an upstream kinase for LKB1 phosphorylation [51, 67] we also observed that LKB1 phosphorylation by zinc could be mediated by PKC, and showed that LKB1 phosphorylation was almost completely attenuated by GF109203X, the specific chemical inhibitor of PKC, (Fig. 3e). However, further studies are needed to clarify the signaling cascade related to LKB1 and CaMKKβ action in zinc neurotoxicity.

In addition to the phosphorylation of LKB1 and AMPK, we observed elevated expression levels of LKB1 and AMPK in zinc-induced neuronal death. In the case of AMPK, the clear increase of AMPK protein levels induced by zinc was preceded by the phosphorylation of AMPK (Figs. 2 and 3). Whereas the noticeable elevation of AMPKα1 and AMPKα2 proteins was detected beginning at 30 min after the exposure of neuronal cultures to zinc, the significant increase of AMPK phosphorylation was observed beginning at 2 h after the onset of zinc toxicity. Furthermore, the levels of AMPK and phosphorylated AMPK proteins were regulated by LKB1, because selective siRNAs for LKB1 almost completely reversed AMPK induction by zinc (Fig. 3c). In the present study, we mainly focused on the phosphorylation of LKB1 and AMPK; however, the regulation of LKB1 and AMPK expression seemed to be crucial for zinc-induced neuronal death. Metabolic stress, such as high AMP levels, activates AMPK, and triggers phosphorylation of the subfamily O of forkhead box transcription factor 3 (FOXO3) at Ser413. Activated FOXO3 can bind and activate its own gene promoter via a positive auto-regulatory feedback loop, which results in the elevation of FOXO3 transcription [68]. Enhanced FOXO3, in turn, induces the master upstream kinase LKB1 that further activates AMPK [68]. Thus, FOXO3 may act as a transcription factor to induce and activate LKB1 and AMPK under metabolic stress. In future studies, we will examine the possibility that FOXO3 regulates *de novo* synthesis of LKB1 or AMPK in zinc-induced neuronal death.

Zinc-induced neuronal death has both necrotic and apoptotic aspects in cortical neuronal cultures, depending on the duration of zinc exposure and magnitude of intracellular zinc loading [18, 19]. Whereas a 10-min exposure of mouse cortical neuron cultures to 400 μM zinc showed more rapid necrosis (data not shown), exposure of mouse cortical neuron cultures to 300 μM zinc for 10 min showed only a mild delay in apoptosis characterized by cell shrinkage, and nuclear condensation and fragmentation (Fig. 1a). Here, we used the apoptotic zinc neurotoxicity model (300 μM zinc for 10 min), because the protective effect of compound C, an AMPK inhibitor, was maximized in this model. Several studies have demonstrated that the pro-apoptotic BH3-only proteins of the Bcl-2 family were induced by AMPK activation in diverse apoptosis models [36, 69, 70]. Interestingly, apoptosis of cerebellar granular neurons in response to excitotoxicity was mediated by rapid AMPK activation and resultant induction of the BH3-only protein Bim [36]. Hence, we examined the AMPK-mediated induction of BH3-only proteins in zinc-induced caspase-3 activation and neuronal death. Consistent with the hypothesis, the noticeable increase in mRNA and protein levels of Bim-EL, Bim-L, PUMA, and Noxa, and the slight elevation of mRNA and protein levels of Bax were detected after the zinc exposure of cortical neuronal cultures (Fig. 5a & b). However, *de novo* synthesis of Bim but not PUMA, Noxa, or Bax, was mediated only by AMPK and LKB1 in zinc-induced cortical neuronal death (Fig. 5d & f). Recently, it was reported that excitotoxicity activated the Bim promoter in a FOXO3-dependent manner, which required direct phosphorylation of FOXO3 by AMPK [69]. To identify the upstream signaling cascade for other BH3-only proteins including PUMA, Noxa, and Bax, other transcription factors closely associated with apoptosis, such as p53, should be evaluated in zinc-induced neuronal death. As our understanding of AMPK regulation expands, our data reveal the tissue- and context-specific regulators that may be targeted for the development of new drugs, or alternative therapies for the treatment of acute brain injury.

## Conclusions

We demonstrated the crucial role of AMPK in zinc-triggered cortical neuronal death. LKB1 activation, rather than increased AMP/ATP ratio or CaMKKβ activation, triggered a rapid activation of AMPK, and resultant induction of the pro-apoptotic BH3-only protein, Bim. Finally, caspase-3-mediated neuronal apoptosis was induced by exposure to excessive zinc in mouse cortical neuronal cultures. Because zinc neurotoxicity is likely a key component in neuronal death related to acute brain injury, the identification of this LKB1-AMPK-Bim signaling cascade in zinc neurotoxicity is critical for the development of new therapeutics for acute brain injury.

## Methods

### Mouse near-pure cortical neuronal cultures

Primary near-pure neuronal cultures were prepared from embryonic mice at 13–14 days as described previously [18]. In brief, triturated cortical cells were seeded onto a poly-D-lysine coated plate (SPL Life Sciences, Gyeonggi-do, South Korea) at seven hemispheres per 24-well plate or nine hemispheres per 6-well plate, respectively. Growth medium consisted of glutamine-free Dulbecco’s modified Eagle medium (DMEM, GibcoBRL, Grand Island, NY, USA) with 25 mM glucose, 44 mM sodium bicarbonate, 2 mM glutamine, 5 % fetal bovine serum, and 5 % horse serum. For pure neuronal cell culture, 10 μM cytosine arabinoside (Sigma, St. Louis, MO, USA) was added at days *in vitro* (DIV) 3. Cultures were incubated at 37 °C in a humidified 5 % CO_2_ atmosphere. This procedure routinely produced near-pure cortical neuronal cultures through DIV1 to DIV7 consisting of > 96 % neurons, < 1 % astrocytes, and < 0.5 % microglia. All experiments were performed at DIV 6–7.

The present study was conducted in accordance with the guidelines for care and use of mice in research and under protocols approved by the Animal Care and Use Committee of The Sejong University.

### Treatment with zinc and chemicals/drugs

Near-pure cortical neuronal cells were washed with Eagle’s minimal essential medium (MEM, GibcoBRL) and chemicals/drugs [Compound C (Dorsomorphin dihydrochloride, Tocris, Bristol, UK), metformin (Sigma), GF109203X (Sigma), or STO-609 (Sigma)] were added 30 min before zinc treatment. Near-pure cortical neuronal cultures were treated with 300 μM zinc in Hank’s balanced salt solution (HBSS, Welgene, Gyeongsangbuk-do, South Korea) supplemented with 1.8 mM CaCl_2_, 1.22 μM MgSO_4_, 3.15 μM MgCl_2_, and 1.94 mM glucose for 10 min. Subsequently, the zinc solution was replaced by MEM and chemicals/drugs were replenished post-treatment.

### Estimation of cell death

Cell death was detected by terminal deoxynucleotidyl transferase-mediated dUTP nick end labeling (TUNEL, *In Situ* Cell Death Detection Kit, Fluorescein; Roche, Indianapolis, IN, USA) and 2 μg/ml Hoechst-33258 staining (Invitrogen, Carlsbad, CA, USA). Cultures were fixed with 4 % paraformaldehyde for 15 min and then permeabilized with 0.1 % Triton X-100 for 10 min at room temperature. TUNEL staining was performed according to the manufacturer’s instructions and was followed by Hoechst staining. Stained cells were analyzed using a digital inverted fluorescence microscope (EVOS Cell Imaging System, Thermo Fisher, Waltham, MA, USA). Quantitative TUNEL-positive cell counting data were based on the ratio of TUNEL-positive to Hoechst-positive cells.

In addition, cell injury was measured by the level of lactate dehydrogenase (LDH) released from the irreversibly damaged or dead cells into the medium [71]. LDH values were normalized to the maximal LDH release (=100 %) after 12 h exposure to 100 μM NMDA in sister cultures.

### Measurements of intracellular AMP and ATP levels

Cortical neuronal cultures were washed trice with cold phosphate-buffered saline (PBS, Welgene) and lysed in 1.4 ml of cold methanol/H_2_O (80 % vol/vol) by vigorous vortexing. The internal standard solution (100 μL of 5 μM glutamine-d_4_; Sigma) was added to the cell extracts. After centrifugation at 14,000 rpm for 10 min, pellets were resuspended in RIPA lysis buffer [50 mM Tris (pH 7.5), 150 mM NaCl, 1 % NP-40, 0.5 % deoxycholic acid, 0.1 % SDS, and 5 mM EDTA with freshly prepared protease/phosphatase inhibitors (2 μg/ml aprotinin, 2 μg/ml leupeptin, 1 μg/ml pepstatin A, 1 mM PMSF, 1 mM Na_3_VO_4_, 5 mM NaF, and 10 mM Na_4_P_2_O_7_)] and the supernatant containing polar metabolites and lipids was collected. Three ml of CHCl_3_/H_2_O (35 % vol/vol) were added to the supernatant and the sample was mixed. The upper aqueous layer (polar metabolites) and lower organic phases (lipids) were separated by centrifugation at 2,000 xg for 15 min. The organic phase was discarded and the aqueous phase was extracted again with 1 ml of CHCl_3_/H_2_O (50 % vol/vol) and then centrifuged at 2,000 xg for 15 min. The aqueous phase was collected and dried using a vacuum centrifuge, and analyzed using liquid chromatography-tandem mass spectrometry [LC-MS/MS; 1290 HPLC (Agilent Technologies, Palo Alto, CA, USA), Qtrap 5500 (AB Sciex, Concord, Ontario, CAN) with a reverse-phase column (Synergi fusion RP 50 × 2 mm, Phenomenex, Torrance, CA, USA)] [72].

### Measurement of in vitro AMPK enzyme activity

AMPK enzyme activity was measured using the CycLex AMPK Kinase Assay Kit (MBL, Nagoya, Japan) and purified recombinant AMPK (A2/B1/G1, Active; MBL), as described previously [73]. In brief, an IRS-1 S789 (AMPK α-2 substrate) pre-coated plate was incubated with 3 ng of active AMPK enzyme with or without 1 μM of zinc chloride in kinase reaction buffer supplemented with 5 μM ATP (according to manufacturer’s protocol for activator screening). To measure AMPK activity, the amount of phosphorylated substrates was detected using anti-phospho-mouse IRS-1 S789 monoclonal antibody followed by horseradish peroxidase-conjugated anti-mouse IgG. Color change was measured as absorbance at 450 nm with a microplate reader (Molecular Devices, Sunnyvale, CA, USA).

### Reverse-transcription polymerase chain reaction (RT-PCR)

Total RNA from cortical neurons was isolated with Trizol (Invitrogen). RNA (2 μg) was reverse transcribed to cDNA and added to the PCR reaction mix (AccuPower PCR premix kit, Bio-Rad, Hercules, CA, USA) according to the manufacturer’s instructions. PCR was performed with *bim* primers (5’-AGCCTGCTGAGAGGCCTCCC-3’ and 5’-GATCCGCCGCAGCTCCTGTG-3’) or *actin* primers (5’-TCTACAAATGTGGCTGAGGAC-3’ and 5’-CCTGGGCCATTCAGAAATTA-3’) for 35 cycles (94 °C for 1 min, 63 °C for 30 s, and 72 °C for 1 min), *noxa* primers (5’-TTGCGCAGCCCGAGTCTTGG-3’ and 5’-GGTTCACTGGCGCGTTCCGA-3’) for 34 cycles (94 °C for 1 min, 60 °C for 1 min, and 72 °C for 1 min), or *puma* primers (5’-CCGACCCTCACCCTGGAGGG-3’ and 5’-AAGTCTCCGACGTCCCCCGG) for 30 cycles (95 °C for 1 min, 59 °C for 1 min, and 72 °C for 1 min). PCR products were electrophoresed on a 2 % agarose gel, stained with ethidium bromide, and then visualized using (BIS 303 PC, DNR Bio-imaging Systems Ltd, Jerusalem, Israel).

### Western blots

Cell lysates were prepared in RIPA lysis buffer containing 50 mM Tris (pH 7.5), 150 mM NaCl, 1 % NP-40, 0.5 % deoxycholic acid, 0.1 % SDS, and 5 mM EDTA with freshly prepared protease/phosphatase inhibitors (2 μg/ml aprotinin, 2 μg/ml leupeptin, 1 μg/ml pepstatin A, 1 mM PMSF, 1 mM Na_3_VO_4_, 5 mM NaF, and 10 mM Na_4_P_2_O_7_). For western blotting of BH3-only Bcl family proteins and caspase-3, a proteasome inhibitor (10 μM MG-132, Sigma) was added to the protein extracts. Thirty micrograms of total protein were electrophoresed on 8 or 15 % polyacrylamide gels and then transferred to PVDF membranes. Anti-phospho-AMPK T183(α-1)/T172(α-2), phospho-AMPK S491(α-2), AMPK α-1, AMPK α-2, Noxa, Puma (Abcam, Cambridge, UK), phospho-AMPK S485(α-1), AMPK α, Bim, caspase-3, cleaved-caspase-3, p-LKB1, or LKB1 (Cell Signaling Technology, Danvers, MA, USA) antibodies were used. Actin (Sigma) was used as the loading control. To visualize the protein bands, enhanced chemiluminescence (iNTRoN Biotechnology, Gyeonggi-do, South Korea) and a bio-imaging system (BIS 303 PC, Philekorea Technology, Daejeon, South Korea) were used. Normalized band intensity was quantified using Image J software.

### Knockdown of LKB1 with siRNA and transfection with cDNA of the active form of AMPKα1 or AMPKα2

Three different predesigned LKB1 (RNA accession number NM_011492.1) siRNA oligonucleotides were purchased from Bioneer (Daejeon, South Korea) (#1, sense 5’- CGACAGAUUAGGCAGCACA-3’ and antisense 5’-UGUGCUGCCUAAUCUGUCG-3’; #2, sense 5’-CUCGUACCUAUCCCACCAA-3’ and antisense 5’- UUGGUGGGAUAGGUACGAG-3’; #3, sense 5’- CCACCGAGGUAAUCUACCA-3’ and antisense 5’-UGGUAGAUUACCUCGGUGG-3’). Non-selective control siRNA was obtained from Invitrogen (catalog number 45–2001). cDNA of the active form of human AMPKα1 or AMPKα2 (Thr172 replaced with aspartic acid) was generously provided by Dr. Ha (Kyung Hee University, South Korea). The transfection of siRNA into mouse cortical neuron cultures was performed using Lipofectamine RNAiMAX (Invitrogen) at DIV 4 according to the manufacturer’s instructions. Lipofectamine 2000 (Invitrogen) instead of RNAiMAX was used for cDNA transfection. After 48 h incubation, zinc solution (300 μM for 10 min) was added to cortical neuronal cultures to induce zinc neurotoxicity and AMPK activation.

### Statistical analysis

A two-tailed *t*-test was used to determine statistically significant differences between groups. Results are presented as mean ± SEM. Statistically significant differences from control are presented as *p < 0.05 and **p < 0.01.

## Abbreviations

AMPK: AMP-activated protein kinase
Bcl-2: B-cell lymphoma 2
BH3: Bcl-2 homology domain 3
CaMKKβ: Ca^2+^/calmodulin-dependent protein kinase kinase β
LDH: Lactate dehydrogenase
LKB1: Liver kinase B1
MO25: Mouse protein 25
nNOS: Neuronal nitric oxide synthase
PARP: Poly(ADP-ribose) polymerase
PKA: cAMP-dependent kinase
PKCζ: Protein kinase C zeta
PUMA: p53 upregulated modulator of apoptosis
STRAD: STE20-Related adaptor protein
